# WOULD INTEGRATING MONOCHROMATIC INFRARED ENERGY INTO THE PHYSICAL REHABILITATION OF ADOLESCENTS WITH PATELLOFEMORAL PAIN SYNDROME HAVE ANY ADVANTAGEOUS EFFECTS? A RANDOMIZED CONTROLLED TRIAL

**DOI:** 10.2340/jrm.v57.42169

**Published:** 2025-03-24

**Authors:** Saud M. ALRAWAILI, Alshimaa R. AZAB, Ragab K. ELNAGGAR, Norah A. ALHWOAIMEL, Nourah BASALEM, Aram A. ALASEEM, FatmaAlzahraa H. KAMEL, Maged A. BASHA, Saleh M. ALORAINI, Walaa E. MORSY

**Affiliations:** 1 Department of Health and Rehabilitation Sciences, College of Applied Medical Sciences, Prince Sattam Bin Abdulaziz University, Al-Kharj, Saudi Arabia; 2 Department of Physical Therapy for Pediatrics, Faculty of Physical Therapy, Cairo University, Giza, Egypt; 3 Department of Physical Therapy, College of Applied Medical Sciences, Qassim University, Buraydah, Saudi Arabia; 4 Department of Physical Therapy for Surgery, Faculty of Physical Therapy, Cairo University, Giza, Egypt; 5 Department of Physical Therapy, El-Sahel Teaching Hospital, General Organization for Teaching Hospitals and Institutes, Cairo, Egypt; 6 Physical Therapy Department, College of Nursing and Health Sciences, Jazan University, Jazan, Kingdom of Saudi Arabia

**Keywords:** monochromatic infrared energy, patellofemoral pain syndrome, physical therapy, rehabilitation, postural control, functional performance, quality of life

## Abstract

**Objective:**

This study aimed to assess and compare changes in pain, balance, functional status, and health-related quality of life between adolescents with patellofemoral pain syndrome undergoing 12 weeks of monochromatic infrared energy application as an adjuvant to physical rehabilitation and those receiving physical rehabilitation alone.

**Design:**

Randomized controlled trial.

**Patients:**

Adolescents aged 15–18.

**Methods:**

46 adolescents were randomly assigned to receive either a standard physical therapy programme or monochromatic infrared energy plus the standard programme. Pain intensity, dynamic postural control, functional status, and health-related quality of life were evaluated pre- and post-intervention.

**Results:**

The study group showed a greater reduction in pain intensity (*p* < 0.001; η^2^ = 0.36), improvement in dynamic postural control, towards the anterior (*p* = 0.002; η^2^ = 0.20), posteromedial (*p* = 0.009; η^2^ = 0.14), posterolateral (*p* = 0.018; η^2^ = 0.12) directions, and composite postural control (*p* = 0.001; η^2^ = 0.24), and enhancement of functional status (*p* = 0.013; η^2^ = 0.13) from the pre- to post-treatment occasion than the control group. Moreover, the study group reported better quality of life: physical health (*p* = 0.035; η^2^ = 0.10), psychosocial health (*p* = 0.005; η^2^ = 0.17), and overall (*p* = 0.001; η^2^ = 0.21).

**Conclusion:**

Monochromatic infrared energy is likely beneficial in adolescents with patellofemoral pain syndrome.

## LAY ABSTRACT

The purpose of this study was to assess and compare changes in pain, balance, functional status, and health-related quality of life between adolescents with patellofemoral pain syndrome undergoing 12 weeks of monochromatic infrared energy (MIRE) application as an adjuvant to physical rehabilitation and those receiving physical rehabilitation alone. A randomized controlled trial included 48 participants aged 15 to 18, who were randomly allocated to either a traditional physical therapy programme or MIRE plus the traditional programme. Key outcomes such as pain, dynamic postural control, functional status, and health-related quality of life were assessed before and after therapy. The MIRE group showed larger gains in pain relief, postural control, functional status, and overall quality of life than the control group. These data indicate that MIRE may be a useful adjunct to physical therapy for improving outcomes in adolescents with patellofemoral pain syndrome.

Patellofemoral pain syndrome (PFPS) is one of the most frequently presenting orthopaedic disorders that affects adolescents and young adults. It is predicted to affect 7% of young active persons and approximately 26% of young athletes (1), with a higher frequency among females (1.5–3 times as frequent as males) (2). Lower extremity weakness, particularly in the quadriceps muscle, malalignment, foot deformities (such as increased subtalar pronation), and tightness of the lateral retinaculum, iliotibial band, and hamstring muscles, are common causes for PFPS (3).

PFPS is typically characterized by diffuse anterior knee pain, retro-patellar pain (behind the kneecap) or peripatellar pain (around the kneecap), and crepitation in the knee joint that is aggravated by certain activities that increase the compressive loading pressures across the patellofemoral joint (these include squatting, extended sitting, running, climbing, and ascending and descending stairs) (4). Alongside pain, PFPS patients have alterations in muscle activity patterns, especially the quadriceps, which lead to improper positioning of the patella; these can influence the ability to maintain balance and motor control (5). As reported in some studies, patients with PFPS demonstrated worse postural control during the balance test on an unsteady platform and the Star Excursion Balance Test (6, 7).

Reduced flexibility in the rectus femoris, hamstrings, and TFL & IT band, combined with increasing pain, may result in a decline in functional status (8). Blond and Hansen has been hypothesized that between 71% and 74% of individuals with PFPS alter their physical activity and functional status due to pain (9). Also, Piva et al. observed that PFPS patients reported low scores on self-reported functional scales (10). The concept of quality of life encompasses many different aspects, including physical, psychological, social, and personal components. A recent study found that PFPS, which encompasses serious medical issues, primarily affects young people and has an influence on the quality of life (11). Silva et al. stated that individuals with PFPS experienced a significant decline in quality of life regardless of how active they were (12).

Physical therapy modalities are essential to treat patients with PFPS to alleviate pain, and to improve or even maintain functional performance (13). Among these modalities, pulsed ultrasound, hot application, and transcutaneous electrical nerve stimulation are commonly used to improve local circulation and decrease pain (14). Monochromatic infrared energy or MIRE is a relatively recent electrotherapy technology. It is a device that emits light with a wavelength of 890 nm, which has been demonstrated to enhance sensation and lessen pain (15). MIRE was approved by the US Food and Drug Administration in 2002 for pain relief for different musculoskeletal cases such as carpal tunnel syndrome, arthritis, and knee osteoarthritis (16).

As many prior studies have shown that MIRE decreases pain and in turn improves muscle performance, balance, and functional abilities in many musculoskeletal disorders, experimental research on its efficacy in adolescents with PFPS is inadequate. This study thus aimed to compare the effects of a 12-week MIRE application combined with physical rehabilitation against physical rehabilitation alone, primarily assessing changes in pain and balance in adolescents with PFPS. Secondarily, this study examined functional status and HRQoL to provide a comprehensive view of treatment outcomes.

## METHODS

### Study design

Between October 2021 and September 2022, this randomized controlled clinical trial was conducted at the outpatient clinic, College of Applied Medical Sciences, Prince Sattam bin Abdulaziz University (PSAU), Saudi Arabia. The PSAU’s Physical Therapy Research Ethics Committee approved the study ethically (RHPT/0021/0026). All procedures were carried out in agreement with the 1975 Helsinki Declaration. Before enrolment, participants and their guardians were fully informed about the study procedures, benefits, and potential risks before signing a consent form. An uninformed independent researcher measured pain, balance, functional status, and quality of life before and after 3 months of treatment. The following identifier has been assigned to the study on ClinicalTrials.gov: NCT05959148.

### Participants

Forty-six patients of both sexes were recruited from physical therapy and orthopaedic clinics where they attend rehabilitation. They were included if aged from 15–18 years, were complaining of pain anterior to the knee joint or retro-patellar during rest that increased with activities such as prolonged sitting, squatting, running, and stair climbing. They were also included if they had PFPS with insidious onset for more than 6 weeks without any traumatic incidence, and they had not participated in a physical therapy programme for the past 3 months. Patients with meniscal tears, cruciate/collateral ligament injury, knee osteoarthritis or rheumatoid arthritis, and a previous patellar dislocation/subluxation, knee ,or hip surgery were all ruled out.

### Assignment procedure

After the initial assessment, enrolled patients were randomly assigned into 2 equal-sized groups. A simple randomization was performed through sealed non-transparent envelopes by an independent researcher. The MIRE group (*n* = 23) received MIRE in addition to the physical therapy exercise programme) and the control group (*n* = 23) received physical therapy exercise programme only.

### Outcome measures

Measurements of pain, balance, functional status, and quality of life were undertaken pre- and post-treatment by an independent researcher who was not aware of the treatment allocation.

### Primary outcome measures

*Pain assessment.* A 10-cm visual analogue scale (VAS) was used to measure the intensity of the pain, with 0 representing no discomfort and 10 being the worst pain experience. The participants were instructed to cut the line at a position that represented the level of pain they felt either at rest or while engaging in an activity (17).

*Dynamic postural control.* To assess dynamic postural control in patients with PFPS, the modified Star Excursion Balance Test (mSEBT) was used as it is functional, valid, easy to apply, and cost-effective (18). A simple instructional video on test procedures was viewed by the patients before the test. The mSEBT was performed by participants standing in the centre of a grid laid out on the floor with 3 reach lines forming a Y with standard tape measures and transparent tape on the floor. The 3 reach lines were named, according to the stance limb, anterior (A), posteromedial (PM), and posterolateral (PL), with 2 angles of 135° (between the A and PM line and between the A and PL line) and one angle of 90° (between the PM and PL line). Participants were instructed to stand on 1 limb and extend as far as they could with the distal portion of the reaching limb pointing in each direction without altering their weight. The distance from the centre of the mSEBT to the point of the foot reach was then measured with a measuring tape (in/cm), and the reaching limb was restored to the starting position. For the purpose of this study, the reach distance in all directions (anterior, posteromedial, and posterolateral) and the composite distance were normalized to the length of the leg and employed for data analysis.

### Secondary outcome measures

*Functional status.* The Arabic version of the Anterior Knee Pain Questionnaire (Kujala Questionnaire) was used to assess functional status (19). It is a 13-item self-reported survey that measures the functional restrictions brought on by patellofemoral pain. Questions reflect pain, aberrant patellar movement, oedema, restricted knee flexion, and quadriceps wasting throughout 6 physical activities, which are sitting, squatting, walking, climbing stairs, jumping, and running. The score ranges from 0 to 100, where 100 indicates no discomfort or functional restriction and 0 indicates complete functional disability. The scale’s test–retest reliability is strong (ICC = 0.86–0.94) and its test validity is moderate (20).

*Quality of life.* The self-report Pediatric Quality of Life Inventory (PedsQL) was used to assess the quality of life. The PedsQL is a 23-item multidimensional evaluation of the health-related quality of life (HRQL) of children and adolescents; it is divided into 4 domains: physical (8 items), emotional (5 items), social (5 items), and school functions (3 items). It has been validated to use with children and adolescents from 2–18 years (21). Each item is scored out of 5 (0 means never, and 4 means almost always). Items are scaled linearly from 0 to 100 (0 = 100, 1 = 75, 2 = 50, 3 = 25, and 4 = 0). In this study, we derived the physical health summary score, which is the sum of the item scores in the physical domain divided by the number of rated items, and the psychosocial health summary score, which is the sum of the item scores in the emotional, social, and school functioning domains, and the overall summary score (the sum of all item scores divided by the number of rated items in all domains). A higher value denotes a higher standard of living.

### Intervention

*Traditional physical therapy programme.* Participants in both groups underwent the physical therapy exercise programme, which consisted of 3 sessions per week for 3 months, each lasting 60 min. The programme’s major goals were to reduce pain, strengthen muscles, improve flexibility, and enhance functional status. The programme includes stretching and strengthening exercises for the muscles surrounding the hip and knee, and balance exercises, as well as electrotherapy (ultrasound and hot application) (22, 23).

*MIRE intervention.* Patients in the MIRE group underwent the MIRE application using an Anodyne Therapy Professional Infrared Therapy System (model 480; Anodyne Therapy LLC, Tampa, FL, USA). Participants were instructed to take off their shoes, socks, and pants before reclining on a regular bed. Eight array diode therapy pads were applied on both knees: 2 over each knee’s medial and lateral aspects and 2 each over its anterior and posterior aspects (24,25), each of which included 60 super-luminescent gallium-aluminium arsenide diodes that produced light energy with an 890-nm wavelength. The device delivered radiant power at a rate of 6.24 W. The treatment was applied for 40 min/session, 3 times a week for 3 successive months.

### Study power and sample size

A preliminary power analysis was conducted using G*Power software (v 3.1.9.2, Dusseldorf, Germany) to determine the appropriate sample size for accurately assessing the intervention effect on pain, which was the primary outcome of interest. The analysis indicated that a total sample size of 38 participants (19 per group) was necessary to achieve a power of 80.49% for detecting an effect size of 0.94, obtained from a preliminary study involving 8 participants who received similar interventions. This large effect size reflects a substantial difference between groups (i.e., a meaningful clinical impact), which could translate to significant improvements in pain levels. The significance level was set at 0.05, and a two-independent-sample *t*-test was employed for analysis. To accommodate a potential dropout rate of 20%, a larger sample size of 46 participants (23 per group) was planned.

### Statistical analysis

SPSS version 24 (IBM Corp, Armonk, NY, USA) was used to complete all statistical analyses. To assess the normality of the data distributions, we employed Q–Q plots for visual inspection, as they provide a reliable method, given the relatively small sample size. A one-way analysis of covariance (ANCOVA) test was utilized to analyse post-treatment differences between the MIRE and control groups regarding dependent measures (i.e., pain, dynamic postural control, functional status, and quality of life). The analysis incorporated pre-treatment values as covariates. Where ANCOVA indicated significant findings, the partial eta-squared (partial η^2^) was utilized to quantify the effect size. The significance level was set at *p* < 0.05.

## RESULTS

### Recruitment and retention of participants

**Fig. 1:**
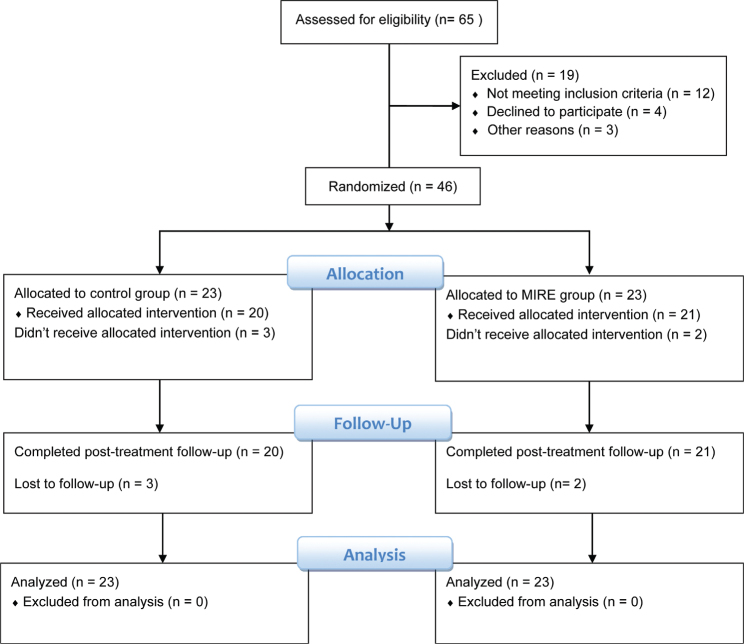
A CONSORT flowchart illustrating participants’ enrolment, randomization, and retention throughout the study.

Fig. 1 shows the CONSORT flow diagram for enrolment, randomization, and retention of the study participants. Sixty-five patients were initially screened for suitability and 46 patients were enrolled and randomized to the study groups. We lost 5 patients before completing the study (2 of the MIRE group and 3 of the control group). They either did not complete the assigned treatment or missed the follow-up assessment for unknown reasons or were unable to comply with the scheduled visits. However, we applied the intention-to-treat principle to the analysis and substituted their missing follow-up data with the pre-treatment observations.

### Baseline characteristics

Table I demonstrates the baseline characteristics of participants of the MIRE and control groups. There were no significant differences between the 2 groups for age, gender distribution, and anthropometric characteristics (weight, height, and body mass index). Also, no significant differences were detected between the 2 groups regarding the clinical attributes (use of painkillers and duration of pain) or frequency of physical activity participation (*p* < 0.05).

**Table I:** Baseline demographic and clinical characteristics of participants of the MIRE and control group

Item	MIRE group (*n* = 23)	Control group (*n* = 23)	*p* - value
Age, years, mean ± SD	16.30 ± 1.15	16.78 ± 1.04	0.15†
Sex (male/female), *n* (%)	13 (56.5)/10 (43.5)	9 (39.1)/14 (60.9)	0.37‡
Weight, kg, mean ± SD	52.78 ± 9.34	55.34 ± 5.62	0.13†
Height, m, mean ± SD	1.56 ± 0.11	1.58 ± 0.08	0.19†
BMI, kg/m2, mean ± SD	21.53 ± 1.33	22.04 ± 1.14	0.18†
Duration of pain, months, mean ± SD	26.22 ± 6.33	24.17 ± 5.49	0.25†
Regular use of painkillers, (Y/N), *n* (%)	7 (30.4)/16 (69.6)	4 (17.4)/19 (82.6)	0.49‡
Regular PA participation, times/week, median (min–max)	2 (1–4)	3 (2–3)	0.11¥

MIRE: monochromatic infrared energy; PA: physical activity; BMI: body mass index; Y/N: yes/no; SD standard deviation.

† Independent t-test,

‡ Fisher’s exact test,

¥ Mann–Whitney *U* test.

### Pain intensity

**Fig. 2:**
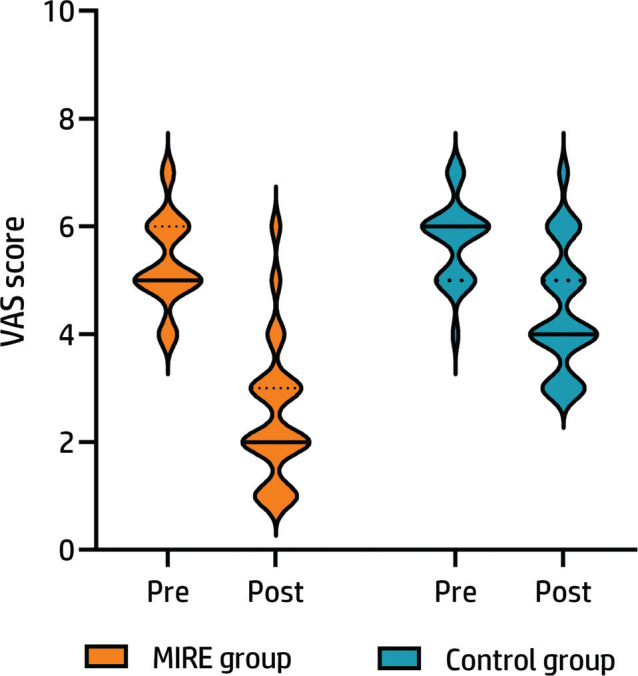
Pre- and post-treatment pain intensity, represented by the patient-reported visual analogue scale scores. The thicker part means the pain scores in that section of the violin have a higher frequency, and the thinner part implies a lower frequency. The continuous lines show the median score, and the dotted lines demonstrate the interquartile ranges.

Fig. 2 displays a violin plot of the pre- and post-treatment distributions and changes in the visual analogue scale scores for the MIRE and control groups. Pre-treatment median (interquartile range) VAS scores for the MIRE and control groups were 5 (5–6) and 6 (5–6), respectively. The post-treatment VAS scores were 2 (2–3) in the MIRE group and 4 (4–5) in the control group. The ANCOVA analysis revealed a significant difference in post-treatment VAS scores between the MIRE group and the control group, after adjusting for pre-treatment scores (F(1,43) = 25.45, *p* < 0.001, partial η^2^ = 0.37). Notably, the MIRE group exhibited lower VAS scores, indicating a greater reduction in pain intensity.

### Postural control

Table II summarizes the dynamic postural control distance changes during the modified Star Excursion Balance Test score (normalized to leg length) in the MIRE and control groups. The results of the ANCOVA indicated a statistically significant difference in post-treatment mSEBT performance, controlling for pre-treatment scores, specifically the distance covered in the anterior distance (F (1,43) = 13.59, *p* < 0.001, partial η^2^ = 0.20), posteromedial distance (F (1,43) = 10.19, *p* = 0.003, partial η^2^ = 0.14), posterolateral distance (F (1,43) = 10.93, *p* = 0.019, partial η^2^ = 0.20), as well as the composite distance (F (1,43) = 23.13, *p* < 0.001, partial η^2^ = 0.35). The MIRE group demonstrated a more substantial increase in the mSEBT distances, reflecting a more pronounced improvement in dynamic postural control.

**Table II:** Post-treatment differences in dynamic postural control as indicated by the modified Star Excursion Balance Test scores (normalized to leg length, %) between the MIRE and control groups, adjusted for pre-treatment scores

Item		MIRE group (*n* = 23) Mean ± SD	Control group (*n* = 23) Mean ± SD	Post-treatment difference	
				Sig.	Partial *η*^2^
ANT distance	Pre	79.44 ± 4.55	78.22 ± 6.12	<.001*	0.24
	Post	86.32 ± 5.48	81.10 ± 5.89		
	MD (95% CI)	6.88 (4.78–8.87)	2.83 (1.44–4.22)		
	Sig.	< 0.001^*^	0.0003^*^		
PM distance	Pre	86.75 ± 4.43	85.58 ± 5.01	0.003*	0.19
	Post	92.99 ± 4.14	89.27 ± 5.10		
	MD (95% CI)	6.24 (4.59–7.88)	2.41 (2.64–4.73)		
	Sig.	< 0.001^*^	< 0.001^*^		
PL distance	Pre	83.56 ± 4.90	80.63 ± 4.39	0.019*	0.20
	Post	89.10 ± 3.84	84.10 ± 4.98		
	MD (95% CI)	5.53 (3.95–7.11)	3.46 (2.70–4.22)		
	Sig.	< 0.001^*^	< 0.001^*^		
Composite distance	Pre	83.25 ± 3.99	81.47 ± 4.71	<0.001*	0.35
	Post	89.47 ± 2.99	84.80 ± 4.59		
	MD (95% CI)	6.23 (4.83–7.61)	3.33 (2.49–4.16)		
	Sig.	< 0.001^*^	< 0.001^*^		

SD: standard deviation; MIRE: monochromatic infrared energy, ANT: anterior, PM: posteromedial, PL: posterolateral, MD: mean difference, CI: confidence interval, Sig: significance level.

* Significant at *p* < 0.05, Partial η^2^: effect size for interaction effect.

### Functional status

**Fig. 3:**
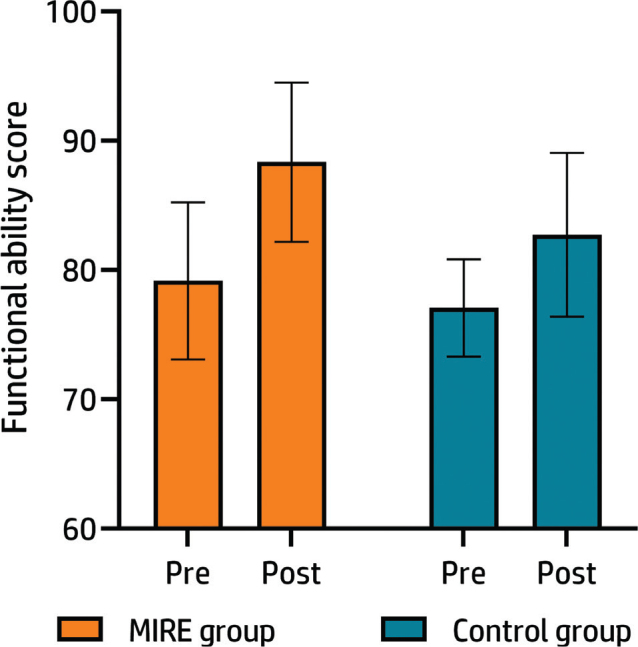
Pre- and post-treatment functional status (Kujala Questionnaire scores). The coloured bars represent the mean score, and the error bars indicate the standard deviations for the means.

Fig. 3 illustrates the pre-to-post changes in the functional status of the MIRE and control groups. Pre-treatment mean (SD) Kujala Questionnaire scores for the MIRE and control group were 79.17 (6.10) and 77.10 (3.76), respectively. The post-treatment scores were 88.35 (6.16) in the MIRE group and 82.74 (6.35) in the control group. The analysis provided evidence of a significant difference in post-treatment scores between the MIRE and control groups, with adjustments made for pre-treatment scores (F (1,43) = 7.65, *p* = 0.008, partial η^2^ = 0.15). The MIRE group reported higher scores, suggesting greater functional enhancement.

### Quality of life

Table III shows the quality-of-life changes (physical, psychosocial, and total scores) in the MIRE and control groups. Table III also shows the quality-of-life changes (physical, psychosocial, and total scores) in the MIRE and control groups. The ANCOVA demonstrated a significant difference in post-treatment physical (F (1,43) = 4.25, *p* = 0.045, partial η^2^ = 0.09), and psychosocial (F (1,43) = 10.55, *p* = 0.002, partial η^2^ = 0.19) health scores, as well as the total quality of life score (F (1,43) = 11.36, *p* = 0.002, partial η^2^ = 0.21) between the MIRE group and the control group, while controlling for pre-treatment scores. The findings indicated that the MIRE group experienced better quality-of-life scores when compared with the control group.

**Table III:** Post-treatment quality-of-life differences (physical, psychosocial, and total summary scores) between the MIRE and control groups, controlled for the pre-treatment scores

Item		MIRE group (*n* = 23)	Control group (*n* = 23)	Post-treatment differences	
				Sig.	Partial *η*^2^
Physical	Pre	75.86 ± 5.83	74.28 ±0.48	0.045*	0.09
	Post	86.10 ± 6.51	80.94 ± 8.048		
	MD (95% CI)	10.23 (8.27–12.19)	6.66 (3.87–9.44)		
	Sig.	< 0.001*	< 0.001*		
Psycho-social	Pre	85.38 ± 5.23	84.85 ± 5.67	0.002*	0.19
	Post	90.57 ± 4.73	87.46 ± 5.77		
	MD (95% CI)	5.18 (3.70–6.67)	2.87 (2.25–3.49)		
	Sig.	< 0.001*	< 0.001*		
Total	Pre	80.62 ± 3.78	79.43 ± 3.33	0.002*	0.21
	Post	88.33 ± 3.77	84.20 ± 5.24		
	MD (95% CI)	7.71 (6.53–8.89)	4.77 (3.45–6.10)		
	Sig.	<0.001*	< 0.001*		

MIRE: monochromatic infrared energy, MD: mean difference, CI: confidence interval, Sig: significance level.

* Significant at *p* < 0.05, Partial *η*^2^: effect size for interaction effect.

## DISCUSSION

PFPS is one of the most common musculoskeletal disorders affecting adolescents, which limits their ability to perform daily functions and engage in physical activities because the pain is excruciating and persists even when they are at rest (26). This study aimed to determine the comparative effects of 12 weeks of MIRE application alongside physical rehabilitation on pain, balance, functional status, and HRQoL in adolescents with PFPS, highlighting differences between the MIRE and control groups. The findings regarding primary outcomes (i.e., pain intensity and dynamic postural control) support the efficacy of the combined MIRE and physical rehabilitation approach. These results demonstrate significant improvements, highlighting the effectiveness of this intervention in addressing key aspects of PFPS. However, the secondary outcomes (functional status and quality of life), being exploratory, indicate potential trends rather than definitive conclusions.

MIRE phototherapy has been found to stimulate the release of nitric oxide (NO) in the endothelium. NO improves microcirculation by relaxing muscles of arteries, veins, and arterioles, leading to vasodilatation and increased blood flow velocity. It is speculated that vessel dilatation, an increase in blood flow rate, enhances the delivery of oxygen and nutrients to the treated area, which in turn decreases pain sensation (27,28). Furthermore, phototherapy can decrease pain by controlling the secretion of inhibitory prostaglandin and cyclooxygenase and enhancing the secretion of normal endorphin and serotonin, and stimulating metabolism (29).

Our results are in line with the results of a study conducted by Rastogi et al., who noted a considerable reduction in the symptoms of neuropathic pain as evaluated by VAS after the application of MIRE 3 times/week for 12 weeks for patients with peripheral neuropathy (30). Also, Robinson et al. found that the overall pain symptoms decreased in ankles and the plantar aspect of feet in patients with type I or type II diabetes after application of MIRE for 30 min 3 times/week for 12 consecutive weeks (31). Further, Bagnato et al. reported an improvement in pain sensation by 25% after the application of monochromatic infrared energy for patients with knee osteoarthritis after 4 weeks and recommended the usage of monochromatic infrared energy as a supplemental therapy for patients with arthritis (32).

There were significant improvements in dynamic postural control, and these improvements may be attributed to pain reduction caused by the application of MIRE. Aminaka and Gribble found that reduction in knee pain in patients with PFPS has a positive impact on Star Excursion Balance performance (33). The improvement in dynamic postural control can further be attributed to the positive outcome of physical exercises on muscle strength and performance, which is in agreement with Salamifar et al., who stated that isotonic and isometric exercises of the lower extremities showed a significant influence on the improved balance (34, 35). Also, Prieto-García et al. suggested that muscle-strengthening exercises for PFPS are effective to reduce pain, improve function (i.e., as indicated by increased Kujala scale score), and promote better quality of life in these patients (36).

Our results also indicated that integrating MIRE into rehabilitation exercises for adolescents with PFPS enhanced their quality of life. Reduced pain perception and greater functional capacity may have been linked to an enhanced quality of life. Our results also corroborated those of van der Heijden et al., who noted simultaneous improvements in pain, functional ability, and quality of life in patients with PFPS following exercise therapy (37). Similarly, Azab et al. found that 12 weeks of exercise has the potential to alleviate pain, increase enhance functional ability, and promote quality of life in adolescents with PFPS (38).

The results of the study should be seen in the context of its limitations, i.e. the absence of long-term follow-up, the relatively small sample size, and the limiting of the sample to the late adolescent stage (15-18 years). Therefore, additional studies that take into consideration the aforementioned limitations are required before we can draw definite conclusions concerning the effect of integrating MIRE into the rehabilitation programme of PFPS patients. This study’s examination of multiple primary outcomes, specifically pain and dynamic postural control, introduces concerns regarding multiplicity. Furthermore, the measurement of dynamic postural control across various stances, each analysed individually, increases the risk of inflating Type I error rates. Although we considered adjusting for multiple testing through methods such as Bonferroni correction, we opted against it due to its conservative nature, which may reduce the power to detect true effects. Therefore, the potential for both Type I and Type II errors remains a significant limitation that warrants further exploration. Future research should aim to refine the analysis of multiple outcomes by exploring alternative statistical approaches that balance the need to control Type I error with the preservation of statistical power. While the trends observed in the secondary measures (specifically functional status and quality of life) suggest potential benefits of the intervention, they should be interpreted with caution. The exploratory nature of these outcomes indicates that further research is necessary to establish their significance and to determine how they may relate to the primary findings.

In conclusion, based on the results of the present study, integrating MIRE into physical rehabilitation programmes for patients with PFPS has the potential to relieve pain, improve dynamic postural control, enhance functional ability, and promote quality of life more favourably than the physical rehabilitation programme alone. Further research is needed to confirm these findings, investigate the underlying mechanisms of MIRE, and develop standardized methods for its use. Future research should also look into the long-term impacts and cost-effectiveness of incorporating MIRE into larger rehabilitation schemes, to ensure its practical and therapeutic utility.
